# Differentiation in Cognitive Abilities Beyond *g*: The Emergence of Domain-Specific Variance in Childhood

**DOI:** 10.1177/09567976251321382

**Published:** 2025-03-18

**Authors:** Lisa Bardach, Robert Kalinowski, Drew H. Bailey

**Affiliations:** 1 Department of Psychology, University of Giessen; 2 Hector Research Institute of Education Sciences and Psychology, University of Tübingen; 3 School of Education, University of California, Irvine; 4 Age of Learning, Glendale, California

**Keywords:** ability differentiation, age differentiation, specific abilities, intelligence, childhood, circumvention of limits, investment theory

## Abstract

Understanding how the structure of cognitive abilities changes depending on age and ability (age and ability differentiation) has critical implications for cognitive-ability assessments and cognitive-developmental theories. Most differentiation research has focused on general intelligence; however, we argue that the investments children make in specific domains and school-taught subjects should rather affect their domain-specific ability structures. Leveraging a representative longitudinal sample of 17,979 U.S. children who were assessed in mathematics, reading, science, working memory, and cognitive flexibility, we found that loadings on a general intelligence factor remained similar, whereas most domain-specific factor loadings increased over time. Hence, age and ability differentiation are conceptually distinct, with the former pertaining to specific abilities and the latter to general intelligence. We find some evidence that domain-specific abilities can compensate for lower general intelligence. Overall, our results encourage a nuanced understanding of children’s cognitive development.

Cognitive abilities play important roles in various aspects of life, encompassing academic achievement, job performance, socioeconomic success, and well-being (Gonzalez-Mulé et al., 2017; Roth et al., 2015). Current conceptualizations of cognitive abilities follow a hierarchical structure. A general intelligence factor (*g*), which pervades all intellectual tasks and accounts for their positive associations (i.e., positive manifold), occupies the vertex of the hierarchy. More specific abilities (e.g., numerical reasoning, memory, visual-spatial thinking) are grouped underneath *g* (Schneider & McGrew, 2018). However, the structure of cognitive abilities is not static, and this fact has vital implications for the accurate assessment of cognitive abilities and for theories of cognitive development (Breit et al., 2022). The term *differentiation* describes how the structure of cognitive abilities depends on ability levels (ability differentiation) and age (age differentiation).

Research on ability differentiation dates to Spearman, whose work compared children who had special-education needs with typically developing children and found that correlations between cognitive-test scores were lower among typically developing children (Spearman, 1927). This phenomenon, named *Spearman’s law of diminishing returns* (or the *ability-differentiation hypothesis*), indicates that the structure of cognitive abilities becomes more differentiated with increasing cognitive ability; hence, the relationship between general intelligence and specific abilities weakens. To put it in factor-analytic terms, we observe lower *g* loadings for individuals with higher levels of general cognitive abilities. Ability differentiation is also consistent with theories that posit that *g* reflects different combinations of cognitive processes in different people, with individuals scoring lower on cognitive tests more constrained by lower-level general processes (Anderson, 2001; Kovacs & Conway, 2019). The ability-differentiation hypothesis has received relatively consistent empirical support, including in samples of children (Blum & Holling, 2017; Breit et al., 2022).

The *age-differentiation hypothesis* (Garrett, 1946) is concerned with changes in the relationships between general intelligence and more specific abilities over the course of development. Two prominent theories make opposite predictions about age differentiation in childhood. According to Cattell’s *investment theory*, fluid intelligence transforms into crystallized intelligence through time, as individuals apply their more basic cognitive abilities to different areas of skill and knowledge in accordance with their interests and environments (Cattell, 1987). Hence, children’s cognitive abilities should become less correlated across time, as children become less constrained by low-level, domain-general abilities and as they become increasingly constrained by domain-specific knowledge, interest, and motivation. Investment theory’s predictions have typically been tested by comparing *g* loadings by age, with decreasing *g* loadings interpreted as support for the theory (Blum & Holling, 2017; Breit et al., 2022).

By contrast, van der Maas et al.’s (2006) dynamic *theory of mutualism* posits that *g* is an epiphenomenon of the reciprocally beneficial interactions of separate cognitive functions, which are initially uncorrelated and become increasingly interrelated in early development. This theory predicts that cognitive abilities become more highly correlated as children develop and that consequently *g* loadings should increase.

In contrast with robust findings of ability differentiation, findings pertaining to the age-differentiation hypothesis in children have been mixed (Breit et al., 2021; Tucker-Drob, 2009). Breit et al.’s (2022) systematic review summarized 21 studies on age differentiation in childhood and adolescence, investigating abilities such as fluid reasoning, crystallized intelligence, visual-spatial thinking, and changes in respective *g* loadings. The results revealed some indications for age dedifferentiation in adolescents (increasing *g* loadings in line with mutualism) and no clear evidence in either direction for children.

We argue that inconsistent prior findings of age differentiation reflect in part a disconnect between the underlying theory and the methods most commonly used to evaluate it. Here, we propose—and demonstrate—that a focus on specific abilities and more narrowly defined content domains in childhood can help amass new knowledge about both age and ability differentiation, expand theories of cognitive development in childhood, and allow for a more appropriate test of some longstanding theoretical predictions.

To illustrate, a novel test of age differentiation that focuses on specific abilities involves testing whether variance specific to content domains changes across childhood. Although *investment theory* predicts increasing differentiation in abilities throughout childhood, analyses of broad-based abilities (e.g., fine motor skills or fluid reasoning) may fall at too general and heterogeneous a level to show this type of pattern; indeed, previous analyses have found little evidence (Cheung et al., 2015) or mixed evidence (Hildebrandt et al., 2016) for emerging domain-specific variances across development (a possible exception is Molenaar et al., 2010, who reported increasing residual test-score variances on some individual cognitive tasks with age in adolescents). Further, some domains may be sufficiently complex that individual differences remain influenced by domain-general abilities long after they are first encountered (Ackerman, 2007). Thus, for broad abilities or complex skill domains, smaller *g* loadings at older ages might not be clearly predicted. A more straightforward prediction of investment theory is that, within a particular content domain, variance should emerge across age and over time as individuals invest *g* to learn specific skills that are afforded by environmental conditions and that individuals are motivated to learn (Murayama, 2022; von Stumm & Ackerman, 2013). For example, children with a strong interest in math who seek out opportunities to practice it spontaneously would not necessarily show substantially higher fine motor skills or fluid reasoning scores across development; however, they likely would show increasing math achievement scores. Hence, as children’s experiences, learning opportunities, and interests have an opportunity to affect specific abilities, the respective variance emerges as a function of these inputs. An important extension of age differentiation research and a well-suited test of investment theory (where does the intellectual investment really go?) therefore involves operationalizing cognitive or skill gains as domain-specific-ability variance that changes by age (i.e., increases in *specific cognitive ability*, or *sca*, loadings) as children enter formal schooling.

Further, a novel test of ability differentiation that accounts for specific abilities involves looking at the differentiation of specific abilities with respect to the level of general ability. Specifically, the *circumvention-of-limits hypothesis* (Hambrick & Meinz, 2011) proposes that high levels of domain-specific knowledge and expertise can circumvent limitations arising from low levels of general cognitive abilities. So far, the circumvention-of-limits hypothesis has been tested by examining interactions between domain-general and domain-specific abilities (e.g., prior experience, domain knowledge) and their effects on domain-specific task performance; such tests have often relied on (relatively small) adult samples. With some exceptions (e.g., Hambrick et al., 2012), tests of the circumvention-of-limits hypothesis have tended to yield null effects: Domain knowledge and cognitive ability are positively related to task performance, with no interaction between them (Glavaš et al., 2023; Hambrick et al., 2018; Hambrick & Meinz, 2011). However, as childhood marks a period in which cognitive abilities undergo major changes (Gualtieri & Finn, 2022; Vergauwe et al., 2021) and likely become more specialized, childhood may be a more sensitive period for testing the circumvention-of-limits hypothesis. In addition, adopting methods used in differentiation research (e.g., nonlinear factor analysis) to examine interactive effects between the general factor (*g*) and specific skill factors (*s*) could provide a strong test of the circumvention-of-limits hypothesis.

This study leverages a large, nationally representative, longitudinal sample of 17,979 U.S. children, who were tested seven times, from kindergarten to fifth grade, on a range of cognitive tasks assessing domain-specific abilities (mathematics, reading, science, working memory, cognitive flexibility). We first tested for the well-established general ability differentiation (upper right quadrant, Fig. 1). We also predicted that the *g*-factor loadings would be consistently large and—because previous findings have been inconsistent—would not vary systematically across waves (upper left quadrant, Fig. 1). Importantly, we hypothesized that the amount of variance accounted for by specific abilities would increase with time, implying that variation in domain-specific abilities increases with the onset of formal schooling (lower left quadrant, Fig. 1). Further, we estimated interactions between general and domain-specific abilities throughout this same age range to allow for the possibility of circumvention of limits (lower right quadrant, Fig. 1).

**Fig. 1.**
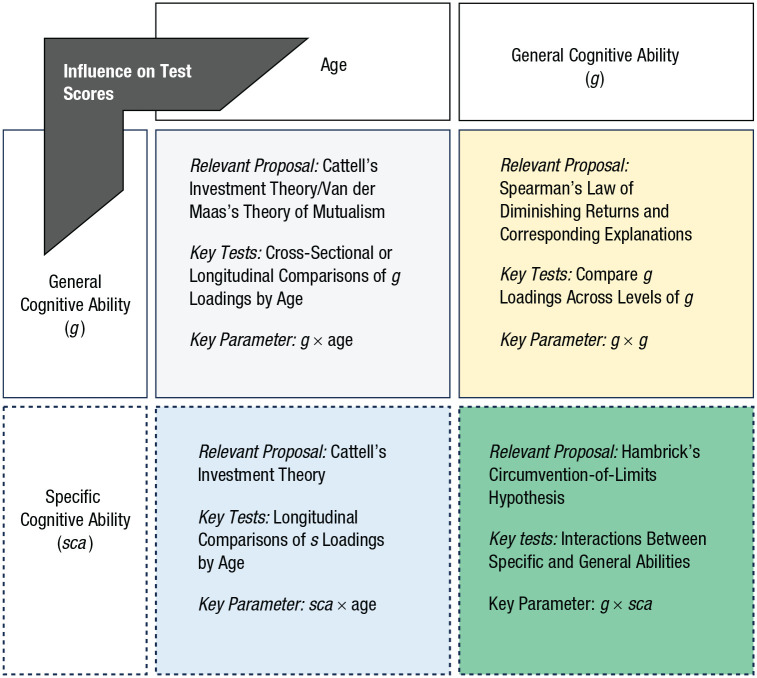
Taxonomy of differentiation: four ways in which differentiation by age and general cognitive ability can be conceptualized and operationalized. General cognitive ability is shown in the upper quadrant, and, as an important extension, specific cognitive ability is shown in the lower quadrant.

## Research Transparency Statement

### General disclosures

**Conflicts of interest:** All authors declare no conflicts of interest. **Funding:** We conducted a secondary data analysis using publicly available data from the Early Childhood Longitudinal Study, Kindergarten Class of 2010–11 (ECLS-K:2011). The ECLS-K:2011 has been funded by the U.S. Department of Education, Institute of Education Sciences, under contract number ED- 04-CO-0059/0023 with Westat. The first author is supported by a research fellowship from the Jacobs Foundation and a fellowship from the Eliteprogramm for Post-docs of the Baden-Württemberg Foundation. **Artificial intelligence:** No artificial-intelligence-assisted technologies were used in this research or the creation of this article. **Ethics:** This article uses secondary data provided by the U.S. Department of Education.

The analysis was registered with the institutional review board (IRB) at the University of California, Irvine, under IRB #5219.

### Study disclosures

**Preregistration:** No aspects of the study were preregistered. **Materials:** The materials consist of items adapted from several copyrighted assessment batteries, which are not publicly available because of copyright restrictions. More information and instructions for accessing these materials are available at the Open Science Framework, or OSF (https://osf.io/brdyt). **Data:** Our study uses publicly available data from the ECLS-K:2011 study, and we share the data set we analyzed at the OSF (https://osf.io/brdyt). **Analysis scripts:** All analysis scripts are publicly available at the OSF (https://osf.io/brdyt). **Computational reproducibility:** The computational reproducibility of the results has been independently confirmed by the journal’s STAR team.

## Method

The current investigation used direct cognitive assessments from child participants of the Early Childhood Longitudinal Study, Kindergarten Class of 2010–2011 (ECLS:K-2011) data set. We used all seven full-sample waves in this analysis. The seven full-sample waves comprised (a) kindergarten entry in 2010, (b) spring of the kindergarten year in 2011, (c) spring of first grade in 2012, (d) spring of second grade in 2013, (e) spring of third grade in 2014, (f) spring of fourth grade in 2015, and (g) spring of fifth grade in 2016. The direct cognitive assessments included tests in five supposedly content-specific domains: (a) numeracy and mathematics (math), (b) vocabulary, print identification, and reading skills (reading), (c) science and general knowledge (science), (d) cognitive flexibility as measured with the Dimensional Change Card Sort (card-sort task; Zelazo, 2006; Zelazo et al., 2013), and (e) working memory as measured with the Numbers Reversed subtest from the Woodcock-Johnson III (reversed-numbers task; Mather & Woodcock, 2001). The analysis was registered with the institutional review board (IRB) at the University of California, Irvine, under IRB #5219.

### Participants

The ECLS-K:2011 collected data from a nationally representative sample of children who entered kindergarten in fall 2010. A multistage sampling design was used involving primary sampling units, schools with probabilities proportional to the number of children, and the selection of a fixed number of children per school. Children who identified as Asian, native Hawaiian, or other Pacific Islander as a single group were oversampled by design. Detailed information on the sampling procedure can be found in the user’s manual for ECLS-K:2011 (Tourangeau et al., 2015; Tourangeau et al., 2017; Tourangeau et al., 2019).

We used all participants who had nonmissing data on at least one cognitive test. This criterion reduced the sample from 18,174 to 17,979. As we conducted a secondary data analysis, no a priori power analysis was applied. However, a recently reported power analysis based on the same analytical approach employed in our study (nonlinear confirmatory factor analysis) yielded a minimum sample size of 1,450 for ability differentiation and 250 for age differentiation to achieve power of > .80 (Breit et al., 2022).

The child participants in our study were on average 67.45 months old when entering kindergarten in 2011 (*SD* *=* 4.47 months). Our sample was 48.9% girls, 46.9% White (non-Hispanic), 13.2% Black (non-Hispanic), 23.2% Hispanic, 8.4% Asian, and 6.1% other racial/ethnic groups.

### Measures

#### Mathematics

The direct mathematics assessment consisted of 81 questions taken directly or adapted from the Test of Early Mathematics Ability–Third Edition (TEMA-3), the Woodcock-Johnson Psychoeducational Battery–Third Edition (WJ-III) Applied Problems Test, and the WJ-III Calculations test. The assessment was designed to measure skills in conceptual knowledge, procedural knowledge, and problem solving and consisted of questions about number sense, properties, and operations; measurement; geometry and spatial sense; data analysis, statistics, and probability; and patterns, algebra, and functions (Tourangeau et al., 2015). Children were first assessed when they entered kindergarten. Eighteen routing items (Stage 1) were administered to each child, the results of which were used to determine the child’s second-stage test difficulty level: low, medium, or high. This scheme was used to gauge children’s abilities as accurately as possible while shortening the administration time. As with all direct cognitive assessments, the math assessment was administered individually by a trained research operative. The child and assessor would typically sit at a desk in a room at the child’s school. Text of test stimuli was presented on a small easel, and this text was read to the child aloud to reduce the impact of reading ability on the math score. Paper, pencil, and blocks were provided to help children answer the questions, but their use was not required for a correct response. Whereas the entire test consisted of 81 questions, each child was administered only the 18 routing items plus the number of items on the child’s Stage 2 test in any one assessment wave. Reliability of the math assessment (Cronbach’s α) was .92, .93, .93, .94, .92, .91, and .92, respectively, for the seven waves (Tourangeau et al., 2019).

#### Reading

The reading assessment was designed to assess children’s language and literacy achievement and included questions measuring basic skills (e.g., print familiarity, letter recognition, word recognition), vocabulary knowledge, and reading comprehension. For the reading-comprehension questions, the children had to identify information specifically stated in the text (e.g., definitions, facts, supporting details). The reading-comprehension questions also required complex inferences to be made within and across texts (Tourangeau et al., 2015). Test items were taken or adapted from the Preschool Language Assessment Scale–Form C: Simon Says and Art Show, the Peabody Individual Achievement Test–Revised (PIAT-R), the Peabody Picture Vocabulary Test–Third Edition (PPVT-III), the Test of Early Reading Ability–Third Edition (TERA-3), and the Test of Preschool Early Literacy (TOPEL). As with the mathematics assessment, a two-stage assessment scheme was used, where 18 routing questions (Stage 1) were used to determine which level of the Stage 2 test (low, middle, or high difficulty) a child would receive. Spanish-speaking children who did not achieve a minimum score on these Stage 1 questions were then administered a similar set of Stage 1 questions in Spanish. Children with a passing score on the Spanish-language version of the basic language questions were then given a Spanish-language version of the Stage 2 test (of appropriate difficulty). Scores on the Spanish-language test were not included in this analysis because the scores were not directly comparable to the English-language test scores. As with all direct cognitive assessments, the reading assessment was administered one-on-one by a trained research operative in a controlled setting, usually at the child’s school. A small easel was used to present visual information to the child. Reliability (Cronbach’s α) of the reading assessment for the seven waves was .92, .94, .95, .90, .86, .87, and .86, respectively (Tourangeau et al., 2019).

#### Science

The science measure was not administered at kindergarten entry. It was first administered to participants in the spring of the kindergarten year. The test included questions about physical sciences, life sciences, environmental sciences, and scientific inquiry. There were 20 items that all children who were administered the science assessment received (a two-stage assessment was not used in this domain). To reduce the likelihood that reading ability would affect the science assessment score, the questions, response options, and any text the children could see were read to them (Tourangeau et al., 2015). This measure was titled Science and General Knowledge for the prior ECLS-K:1998 study. Reliability (Cronbach’s α) of the science assessment for the six waves was .73, .84, .85, .83, .82, and .86, respectively (Tourangeau et al., 2019).

#### Executive functions—cognitive flexibility

The Dimensional Change Card Sort (DCCS) task (Zelazo, 2006) was included as a measure of cognitive flexibility. In kindergarten and first grade, children were presented with a series of cards, each depicting either a red boat or a blue rabbit, and asked to sort these cards into two trays according to specific criteria. In the first set, children were asked to sort cards by color (Color Games). In the second set, the sorting rule was changed, and children were asked to sort by shape (i.e., boat or rabbit; Shape Games). If four of six of these post-rule-change cards were sorted correctly, then children were administered a third set that they would sort by the color rule if the image had a black border and by the shape rule if it did not (Border Games). The total score, which was used in our analyses, reflected children’s performance across the Color, Shape, and Border Games in terms of the number of items that a child sorted accurately (composite score, with a potential range of 0 to 18). Because this physical version would have been too easy for most of the children from second grade on, children completed a new, age-appropriate, computerized version of the DCCS. Here, the “cards” were presented on a computer screen. Children had to sort these cards into virtual piles on the screen by using keys on the keyboard to indicate where to put each card (Zelazo et al., 2013; Tourangeau et al., 2017). This updated version was more challenging because the sorting rule could change at any time, with one rule being dominant (appearing for many more trials) and the switched rule being nondominant. The updated version also measured both speed and accuracy, an appropriate change according to the test developer because older children will sacrifice speed in order to sort cards correctly. The scores for the computerized version had a potential range from 0 to 10. In the computation of the scores, weight was given to accuracy (0 to 5 units) and reaction time (0 to 5 units). Accuracy was considered first, and if a child achieved an accuracy rate of less than or equal to 80%, the child’s overall computed score was based entirely on accuracy. If the accuracy rate was above 80%, the child’s overall computed score was equal to the child’s accuracy score plus the child’s reaction time score. The reaction time score was derived from the child’s reaction time on correct nondominant trials (see Tourangeau et al., 2017, section 3.2.1, for the full formula). Because of the changes in the task and the scoring, change in growth on this task between the first- and second-grade waves should not be included in analyses.

#### Executive functions—working memory

The WJ-III Numbers Reversed task (Mather & Woodcock, 2001) was used to assess working memory. It is an orally administered backwards-digit-span task, where, if the task administrator were to say, “3, 5,” then the child would be expected to respond, “5, 3.” Children were first administered five two-number sequences. If they responded to at least three two-number sequences correctly, they would then be presented with a set of three number sequences. The administration procedure continued in a similar fashion but with longer number sequences until the child answered a total of three items at the same level incorrectly or until the child had been administered all the items. For this analysis, we used the *W* score, a type of standardized score from a special transformation of the Rasch ability scale. *W* scores provide a common scale of equal intervals that represent both children’s ability and item difficulty (Tourangeou et al., 2019).

#### Scoring

In the ECLS-K:2011 study, *item response theory* procedures were used to develop the scores for the math, reading, and science measures. This method makes it possible to compare a score for any child in any domain with any other child in the same domain, even if the two children were not administered the same questions as part of the assessment. Common items across all tests (the routing items, plus items that were common across the different difficulty levels of the Stage 2 tests) were used to calculate scores for all the children on the same scale. This analysis makes use of the theta scores provided in ECLS-K:2011. The theta scores provide an estimate of a child’s latent ability on the basis of the questions the child was actually administered. This score is most appropriate when investigating the development of latent abilities and was selected for this study, as the development of these domain-specific abilities was central to our research questions. More information about the psychometric properties of the assessments can be found in the ECLS-K:2011 user’s manual (Tourangeau et al., 2015) and ECLS-K:2011 Kindergarten Psychometric Reports (e.g., Najarian et al., 2020).

### Statistical analyses

**Fig. 2.**
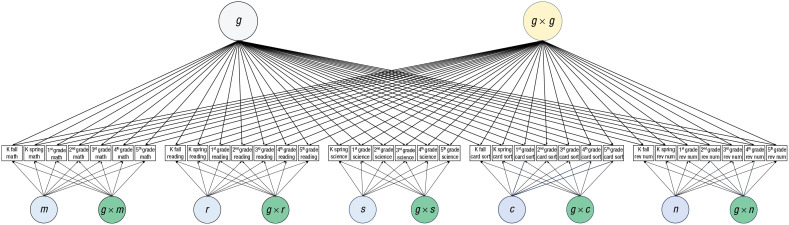
Model displaying general and domain-specific cognitive abilities and their interactions. Observed measures are shown as boxes and are grouped by domain-specific domain. From left to right, the groups of boxes represent measures of math (*m*), reading (*r*), and science (*s*); the card-sort task (c, cognitive flexibility) (*c*); and the reversed-numbers task (*n*, working memory), respectively. K = kindergarten; *g* = general factor.

All analyses were conducted with *Mplus* (Version 8.4; Muthén & Muthén, 2012–2019) using the robust maximum likelihood estimator (MLR) and full-information maximum-likelihood estimation to handle missing data. We analyzed the data using nonlinear factor-analytic methods (see Fig. 2 for the structural model). A basic (linear) factor model assumes that individuals’ abilities are related to their performance on a given outcome to the same extent irrespective of ability level or the level of some other variable; however, it is exactly this assumption that is called into question by differentiation hypotheses. Nonlinear factor models represent structural interrelations as occurring within a single group and along continuous dimensions but in nonlinear and interactive ways (Tucker-Drob, 2009). We used Monte Carlo estimation for the nonlinear factor model and specified 10,000 integration points to achieve sufficient numerical precision and ensure the robustness of the results. Because running our complex model was computationally very intensive, we first set up a nonlinear factor model using the *Mplus* default number of integration points to obtain starting values. Starting values from this model were used for the final model with 10,000 integration points.

Because absolute-fit statistics are not available for nonlinear models or for Monte Carlo integration, we estimated a linear model, omitting all latent variable interactions with an estimator capable of producing fit statistics (see also, e.g., Breit et al., 2021; Tucker-Drob, 2009). The model was otherwise identical to the model in Figure 2. We also compared information criteria—the Akaike information criterion (AIC) and the Bayesian information criterion (BIC)—between the model without interactions and the nonlinear model, with lower values indicating a better trade-off between model fit and complexity. Finally, we conducted a chi-square model-comparison test by using the −2 log likelihood (−2LL) of the nonlinear models compared to that of the linear model and using the additional number of parameters as degrees of freedom for the test (see Tucker-Drob et al., 2011, for an example).

For our study, we extended nonlinear factor analysis (e.g., Breit et al., 2021; Tucker-Drob, 2009) into a longitudinal data analysis (Fig. 2; see below for details on how the longitudinal component was implemented). The model was set up as a bifactor model. Although a variety of factorial models have been designed to represent the structure of cognitive ability, with respect to models that contain *g*, bifactor and higher-order models are often contrasted (Beaujean, 2015). In a bifactor model, one general factor (*g*) influences all observed variables, and there are also group factors (which we call *specific cognitive ability*, or *sca*, factors here) for each domain that are nested in the general factor and that are estimated from the covariance remaining among the observed variables (after accounting for *g*). The factors do not depend on each other, and the influence of a change in the level of *g* on the observed variables is independent of changes in levels of any specific cognitive-ability factors (and vice versa). In comparison, a higher-order model includes one first-order model for each specific domain and a general second-order factor (*g*) that influences all first-order factors. In the higher-order model, shared variance among observed variables contributes to the formation of the specific ability factors but does not contribute to form *g* (Beaujean, 2015; Molenaar et al., 2010). In research on cognitive differentiation adopting a factor-analytical approach, another model that is commonly used is the one-factor model, in which manifest test scores (which often represent different specific cognitive abilities) load on a *g* factor (see, e.g., Breit et al., 2021; Tucker-Drob, 2009).

To summarize, because the bifactor model allows us to estimate the amount of variance explained by domain-specific ability factors and by *g* independently, it was particularly well suited for the present study aiming to investigate cognitive differentiation at both the level of *g* and the five *sca* factors. At the same time, it must be kept in mind that the approach of the current study thus differs from most previous research on cognitive differentiation using factor-analytical approaches.

There was one test score for each domain for each wave; hence, we used the test scores as indicators for the *sca* factors and for *g*. Specifically, in our bifactor model, all tests (i.e., mathematics, reading, science, working memory, and cognitive flexibility) for all seven waves were regressed on the *g* factor, and each test score was also loaded on one of five domain-specific ability factors unique to each domain. That is, math test scores for the seven waves loaded on a latent math ability factor, *m*; reading test scores for the seven waves loaded on a latent reading-ability factor, *r*; and so on (see Fig. 2). The variance of each of these domain-specific ability factors was set to 1, and the five domain-specific ability factors were constrained to be orthogonal to each other and to *g*. All *g* and *sca* loadings were freely estimated (i.e., there were no constraints set on the loadings). Because the data set was very large and we made many statistical comparisons, we set the alpha values to .01. Standard errors were clustered at the first-grade school level.

We next describe how we tested the hypotheses of the current study.

#### Test of ability differentiation

The *general-ability-differentiation hypothesis* proposes that the structure of intelligence becomes more differentiated (i.e., decreases in *g* loadings) with increasing cognitive abilities. Following prior research on ability differentiation using nonlinear factor analysis, we first created a quadratic factor by multiplying *g* by itself (*g* × *g*). All test scores were then regressed on *g* × *g*. The loadings of abilities on *g × g* address ability differentiation in *g*: If the relationships between abilities and test scores differ by location on the ability spectrum, then the quadratic loadings should differ from zero. Hence, depending on the direction of the quadratic effect, the effect of *g* should be stronger or weaker for larger values of *g*. The ability-differentiation hypothesis is supported if quadratic coefficients are negative, indicating diminishing returns for test performance with higher levels of *g* (the *g* factor accounts for decreasing amounts of variance in test scores at increasing ability levels; e.g., Breit et al., 2022; Molenaar et al., 2010; Tucker-Drob, 2009).

#### Test of age differentiation through the early school years

We next investigated age differentiation in *g* (i.e., *general age differentiation*) by comparing *g* loadings across waves. Loadings of the test scores (mathematics, reading, science, etc.) on the *g* factor represent estimates of the contribution of *g* to test scores in each wave across childhood. We predicted that the *g*-factor loadings would be consistently large, and (in line with prior inconsistent findings) should not vary much across waves. With respect to domain-specific abilities, we compared *sca* loadings (loadings of domain-specific test scores on their respective domain-specific ability factor) across waves. These loadings were estimates of the contribution of domain-specific abilities on test scores in each wave across childhood (*domain-specific age differentiation*).

In line with investment theory, the amount of variance accounted for by specific abilities should increase with time, implying that variation in domain-specific abilities increases with the onset of formal schooling. To provide a formal test of changes in *g* and *sca* loadings, we interpreted overlaps in confidence intervals (CIs) between the loadings of each wave and the preceding one. The CIs for *g* loadings should overlap, whereas there should be significant increases for *sca* loadings over time, as indicated by nonoverlapping CIs.

#### Circumvention-of-limits hypothesis

**Fig. 3.**
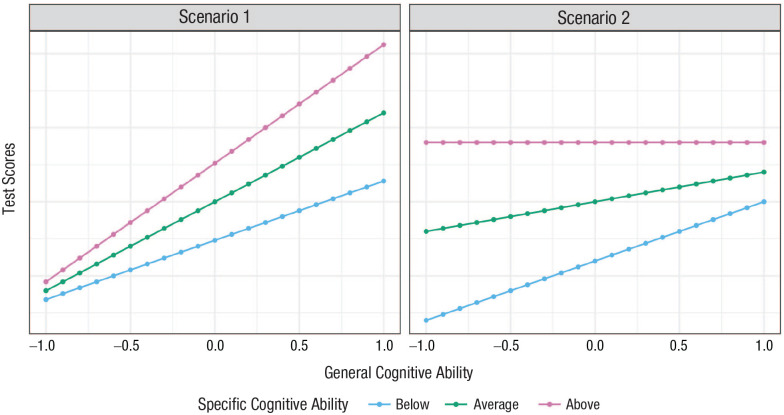
Examples of negative and positive interactions between general cognitive abilities (*g*) and specific cognitive abilities (*sca*).

The *circumvention-of-limits hypothesis* states that high levels of domain knowledge have the potential to enable circumvention of performance limitations that are associated with general cognitive abilities (e.g., Hambrick et al., 2018). In our study, the circumvention-of-limits hypotheses was tested by regressing each test score (i.e., the test scores for mathematics, reading, science, cognitive flexibility, and working memory for each wave) on a general and domain-specific factor interaction (*g* × *sca* interaction): an interaction between the respective domain-specific skill factor *sca* (the latent mathematics factor, the reading factor, etc.) and *g*. If *g* and *sca* are complements, then *g* × *sca* loadings should be small and positive. If, however, children’s domain-specific abilities can stand in for abstract reasoning (i.e., if *g* and *sca* can substitute for each other), then the *g* × *sca* loadings should be small and negative (circumvention-of-limits hypothesis). Figure 3 (right side) illustrates such a compensatory effect in line with the circumvention-of-limits hypothesis. In contrast, on the left side of Figure 3, we show how the interaction between general and specific cognitive abilities might look if general and specific cognitive abilities complement each other in that higher levels of general cognitive abilities allow children to better utilize domain-specific abilities.

#### Robustness checks

In a subsequent model, we added autoregressive effects to the model, connecting different waves of the same domain-specific tests. This model served to check for whether our findings were robust to the addition of autoregressive paths, which likely contribute to the stability among achievement tests in the early school years (Bailey et al., 2014; Wan et al., 2023).

## Results

Descriptive statistics, including information on missing data, and bivariate correlations between all variables used in our analysis can be found in the Supplemental Material available online (Table S1 and Fig. S1, respectively).

We first estimated a model that omitted all latent variable interactions but that was otherwise identical to the model in Figure 2. The fit of this model was acceptable, χ^2^(493) = 28,297.915, *p* *<* .001; comparative fit index (CFI) = .917, root-mean-square error of approximation (RMSEA) = .056. We thus proceeded to estimate the nonlinear model represented in Figure 2 (for which no absolute fit indices were available). Figure 3 and Table 1 present standardized *g*, *sca*, *g × g*, and *g* × *sca* loadings of each of the five test scores in each wave. Each subject domain in Figure 3 is graphed separately for clarity, and identical scales were used for all graphs to aid visual comparison. Model fit indices for this nonlinear model were reduced (AIC = 1,349,587.970, BIC = 1,351,178.550) in comparison with the linear model without the latent variable interactions (AIC = 1,358,255.719, BIC = 1,359,316.106). Results from a chi-square model-comparison test indicated that the fit of the nonlinear model was significantly better than that of the linear model χ^2^Δ(*df*) = 8803.75(68), *p* < .001.

**Table 1.** Standardized Parameter Estimates: Age Differentiation in g (Longitudinal Comparisons of *g*-Factor Loadings by Age), Age Differentiation in Specific Cognitive Abilities (Longitudinal Comparison of *sca* Loadings by Age), Ability Differentiation (*g* × *g* Interactions), and Tests of the Circumvention-of-Limits Hypothesis (*g* × *sca* Interactions)

Model parameters							
Tested domain	Kindergarten entry	Kindergarten spring	First grade spring	Second grade spring	Third grade spring	Fourth grade spring	Fifth grade spring
Math							
*g*	**0.880 (0.003)**	**0.892 (0.003)**	**0.885 (0.004)**	**0.877 (0.004)**	**0.855 (0.005)**	**0.838 (0.005)**	**0.843 (0.005)**
*sca*	**−0.031 (0.011)**	**0.047 (0.010)**	**0.180 (0.010)**	**0.307 (0.010)**	**0.389 (0.009)**	**0.409 (0.009)**	**0.379 (0.009)**
*g × g*	0.015 (0.008)	**−0.060 (0.006)**	**−0.128 (0.006)**	**−0.117 (0.006)**	**−0.092 (0.006)**	**−0.128 (0.007)**	**−0.120 (0.007)**
*g* × *sca*	**0.164 (0.010)**	**0.156 (0.010)**	**0.121 (0.012)**	0.012 (0.011)	**−0.055 (0.010)**	**−0.095 (0.010)**	**−0.112 (0.012)**
Reading/vocabulary							
*g*	**0.814 (0.005)**	**0.820 (0.009)**	**0.833 (0.005)**	**0.827 (0.004)**	**0.841 (0.004)**	**0.837 (0.004)**	**0.828 (0.004)**
*sca*	−0.009 (0.017)	**0.115 (0.016)**	**0.278 (0.011)**	**0.384 (0.009)**	**0.370 (0.007)**	**0.400 (0.008)**	**0.373 (0.008)**
*g × g*	**0.095 (0.006)**	**−0.041 (0.006)**	**−0.146 (0.006)**	**−0.081 (0.006)**	−0.015 (0.006)	−0.016 (0.007)	−0.001 (0.006)
*g* × *sca*	**−0.080 (0.016)**	**−0.133 (0.019)**	**−0.174 (0.013)**	**−0.210 (0.010)**	**−0.122 (0.010)**	**−0.084 (0.012)**	**−0.047 (0.010)**
Science							
*g*	†	**0.710 (0.006)**	**0.774 (0.006)**	**0.798 (0.005)**	**0.806 (0.005)**	**0.779 (0.005)**	**0.783 (0.005)**
*sca*	†	**0.382 (0.009)**	**0.428 (0.008)**	**0.432 (0.008)**	**0.435 (0.008)**	**0.435 (0.009)**	**0.400 (0.010)**
*g × g*	†	−0.013 (0.008)	**−0.039 (0.008)**	**−0.053 (0.008)**	**−0.050 (0.008)**	**−0.091 (0.008)**	**−0.094 (0.008)**
*g* × *sca*	†	**−0.125 (0.009)**	**−0.155 (0.009)**	**−0.156 (0.009)**	**−0.153 (0.008)**	**−0.169 (0.009)**	**−0.162 (0.009)**
Card-sort task							
*g*	**0.441 (0.010)**	**0.436 (0.009)**	**0.451 (0.009)**	**0.536 (0.008)**	**0.504 (0.009)**	**0.480 (0.010)**	**0.465 (0.010)**
*sca*	**0.081 (0.028)**	**0.103 (0.026)**	**0.114 (0.016)**	**0.388 (0.011)**	**0.474 (0.010)**	**0.482 (0.012)**	**0.447 (0.012)**
*g × g*	**−0.075 (0.009)**	**−0.085 (0.008)**	**−0.129 (0.011)**	**−0.147 (0.008)**	**−0.162 (0.009)**	**−0.164 (0.010)**	**−0.143 (0.013)**
*g* × *sca*	**−0.084 (0.032)**	**−0.121 (0.031)**	**−0.118 (0.021)**	**−0.173 (0.012)**	**−0.207 (0.013)**	**−0.162 (0.017)**	**−0.091 (0.020)**
Reversed-numbers task							
*g*	**0.716 (0.005)**	**0.711 (0.005)**	**0.652 (0.007)**	**0.607 (0.008)**	**0.593 (0.009)**	**0.592 (0.008)**	**0.601 (0.009)**
*sca*	**0.082 (0.010)**	**0.126 (0.009)**	**0.259 (0.011)**	**0.430 (0.009)**	**0.512 (0.009)**	**0.542 (0.009)**	**0.508 (0.009)**
*g × g*	**0.107 (0.007)**	−0.016 (0.009)	**−0.122 (0.007)**	**−0.125 (0.008)**	**−0.104 (0.009)**	**−0.088 (0.010)**	**−0.076 (0.010)**
*g* × *sca*	**0.047 (0.007)**	**0.039 (0.008)**	0.021 (0.011)	−0.013 (0.012)	0.022 (0.013)	0.027 (0.013)	**0.054 (0.013)**

Note: ^†^The science test was not administered at Kindergarten entry. Values in bold indicate *p* *<* .01. Standard errors are given in parentheses. Errors were clustered at the first-grade school level. The full model is given in Figure 1. *g* *=* the first extracted factor (the general factor); *sca* *=* the second factor, uncorrelated with *g* (the specific skill factor unique to each tested domain); *g × g* = the *g*-by-*g* interaction; *g* × *sca* = the *g*-by-*sca* interaction, with the *sca* factor varying by the tested domain.

**Fig. 4.**
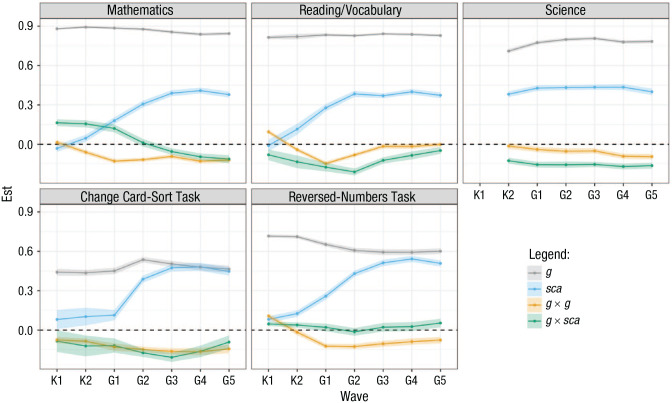
Model estimates (including confidence intervals) of *g*, *sca*, *g* × *g*, and *g* × *sca* loadings on five content-specific domains. The given estimates are for the latent variables *g* and *sca* and the latent variable interactions *g × g* and *g* × *sca* for each content-specific domain. The *x*-axes represent the assessment waves: K1 = Kindergarten entry, K2 = Kindergarten spring, G1-G9 = first grade through ninth grade assessments). The *y*-axes show standardized estimates. *g* = the general factor; *sca* *=* the specific skill factor (unique to each tested domain); *g × g* = the *g*-by-*g* interaction; *g* × *sca* = the *g*-by-*sca* interaction, with the *sca* factor varying by the tested domain.

Loadings of *g × g* were mostly small and negative (see Fig. 4). The small, negative coefficients on the quadratic term indicated diminishing returns at higher levels of *g* on test performance, supporting the general-ability-differentiation hypothesis. For mathematics, no significant *g × g* interaction was obtained at kindergarten entry, but from the kindergarten spring assessment on, all *g × g* loadings were negative and statistically significant. Similarly, *g × g* loadings were not significant for the first science assessment (kindergarten spring) and negative and statistically significant for the remaining waves. For reading, the coefficients were positive and significant at kindergarten entry, then significant and negative from kindergarten spring to second-grade spring, and nonsignificant for the last three waves. For cognitive flexibility (card-sort task), the *g × g* interactions were all significant and negative. For working memory (reversed-numbers task), they were negative and significant, except for kindergarten entry (positive and significant) and kindergarten spring (not statistically significant). Overall, of the 34 estimates, two were positive (both of these in the kindergarten-entry wave), six were not significantly different from zero, and 26 were negative.

Loadings on the *g* factor, representing estimates of the contribution of *g* to test scores in each wave (general age differentiation), were consistently moderate to high and significant and positive. For all tests in all waves, the *g* loadings ranged from .44 to .89, with strong differences between domains (highest *g* loadings for mathematics, followed by reading). However, no clear pattern of decreasing or increasing *g* loadings favoring age differentiation or dedifferentiation, respectively, was obtained (see Fig. 3). Table 2 displays all *g* loadings with 99% CIs.

**Table 2.** Standardized Parameter Estimates with 99% Confidence Intervals for Longitudinal Comparisons of *g* and *sca* Loadings by Age

Model parameters							
Tested domain	Kindergarten entry	Kindergarten spring	First grade spring	Second grade spring	Third grade spring	Fourth grade spring	Fifth grade spring
Math							
*g*	**0.880** [0.871, 0.888]	**0.892** [0.884, 0.901]	**0.885** [0.875, 0.895]	**0.877** [0.866, 0.888]	**0.855** [0.843, 0.867]	**0.838** [0.825, 0.850]	**0.843** [0.831, 0.855]
*sca*	**−0.031** [–0.058, −0.004]	**0.047** [0.020, 0.074]	**0.180** [0.154, 0.206]	**0.307** [0.281, 0.333]	**0.389** [0.366, 0.413]	**0.409** [0.386, 0.432]	**0.379** [0.356, 0.403]
Reading/vocabulary							
*g*	**0.814** [0.800, 0.827]	**0.820** [0.798, 0.843]	**0.833** [0.821, 0.845]	**0.827** [0.816. 0.838]	**0.841** [0.831, 0.850]	**0.837** [0.826, 0.848]	**0.828** [0.817, 0.839]
*sca*	−0.009 [−0.053, 0.036]	**0.115** [0.073, 0.157]	**0.278** [0.250, 0.307]	**0.384** [0.361, 0.408]	**0.370** [0.351, 0.389]	**0.400** [0.379, 0.422]	**0.373** [0.351, 0.395]
Science							
*g*	†	**0.710** [0.694, 0.727]	**0.774** [0.759, 0.788]	**0.798** [0.784, 0.812]	**0.806** [0.793, 0.819]	**0.779** [0.765, 0.793]	**0.783** [0.769, 0.797]
*sca*	†	**0.382** [0.359, 0.404]	**0.428** [0.407, 0.450]	**0.432** [0.412, 0.451]	**0.435** [0.415, 0.455]	**0.435** [0.412, 0.458]	**0.400** [0.375, 0.425]
Card-sort task							
*g*	**0.441** [0.415, 0.467]	**0.436** [0.413, 0.459]	**0.451** [0.426, 0.475]	**0.536** [0.514, 0.557]	**0.504** [0.481, 0.527]	**0.480** [0.455, 0.505]	**0.465** [0.438, 0.492]
*sca*	**0.081** [0.008, 0.154]	**0.103** [0.036, 0.171]	**0.114** [0.074, 0.154]	**0.388** [0.361, 0.416]	**0.474** [0.447, 0.501]	**0.482** [0.451, 0.512]	**0.447** [0.415, 0.478]
Reversed-numbers task							
*g*	**0.716** [0.704, 0.729]	**0.711** [0.698, 0.725]	**0.652** [0.635, 0.670]	**0.607** [0.587, 0.627]	**0.593** [0.571, 0.615]	**0.592** [0.571, 0.614]	**0.601** [0.579, 0.623]
*sca*	**0.082** [0.056, 0.107]	**0.126** [0.101, 0.150]	**0.259** [0.231, 0.286]	**0.430** [0.406, 0.454]	**0.512** [0.487, 0.536]	**0.542** [0.518, 0.566]	**0.508** [0.484, 0.531]

Note: ^†^The science test was not administered at Kindergarten entry. Values in bold indicate *p* *<* .01. Standard errors were clustered at the first-grade level. *g* *=* the first extracted factor (the general factor); *sca* *=* the second factor, uncorrelated with *g* (the specific skill factor unique to each tested domain).

Inspecting how the *g* loadings changed from each wave to the next indicated overlapping CIs of the *g* loadings for all domains, with a few (unsystematic) exceptions: The *g* loadings differed significantly for science for kindergarten spring and first grade (higher in first grade), for cognitive flexibility (card-sort task) for first grade spring and second grade (higher in second grade), and for working memory for kindergarten spring and first grade spring (lower in first grade), and for first grade and second grade (lower in first grade).

Loadings for *sca* were, on average, lower than *g* loadings, ranging from −.03 to .54. However, for most domains, loadings on the *sca* factors, representing estimates of the contribution of specific cognitive abilities to the respective test scores in each wave (specific age differentiation), descriptively showed increases from kindergarten entry to fourth grade before dropping in fifth grade (see Fig. 4). Specifically, for working memory (reversed-numbers task) and cognitive flexibility (card-sort task), *sca* loadings were always significant and positive, and gradually increased until Grade 4, with a slight decrease in Grade 5. For reading, *sca* loadings were not statistically significant at kindergarten entry but became positive from kindergarten spring onward, and they increased between kindergarten spring and second grade, with a slight drop in third grade followed by an increase in fourth grade. Then, as for all domains, there was a drop in fifth grade. Loadings for *sca* for mathematics were significant and negative at kindergarten entry, and then became significant and positive with increasing *sca* loadings from first grade to fourth grade. For science, which was, however, not assessed at kindergarten entry, the pattern was less pronounced.

Comparing overlaps in 99% CIs for each wave and the immediately following one revealed that for mathematics, the *sca* loadings significantly increased over the first five waves, that is, from kindergarten entry to third grade spring. The *sca* loading for the fourth grade did not differ significantly from the third-grade loading, and the fifth-grade loading did not differ significantly from the fourth-grade loading; however, both the fourth and fifth grade *sca* loadings were higher than the second-grade loading (and the loadings of the waves preceding second grade). For reading, significant increases for the first four waves (kindergarten entry to second grade) were documented, whereas the third-grade *sca* loading, fourth-grade *sca* loading, and fifth-grade *sca* loading did not differ significantly from the respective previous wave. As can also be seen in Figure 4, the science *sca* loadings did not differ much across time, except for a significant increase between kindergarten spring and first grade spring. For cognitive flexibility (card-sort task), the *sca* loadings significantly increased between first grade and third grade. Also, the *sca* loading for the fourth grade was significantly larger than the *sca* loading for the second wave and thus also for the waves preceding second grade (but not significantly larger than the third-grade *sca* loading). Last, for working memory (reversed-numbers task), the *sca* loadings significantly increased from kindergarten spring to third grade. In addition, the *sca* loadings for working memory in fourth and fifth grade were significantly larger than those in second grade (and in all waves prior to second grade).

Finally, addressing the general and specific factor interaction, the pattern of *g* × *sca* loadings was negative and significant for all waves for reading, science, and cognitive flexibility (card-sort task), in line with the circumvention-of-limits hypothesis. In mathematics, the *g* × *sca* loadings were significant and positive from kindergarten entry to first grade, statistically nonsignificant in second grade, and significant and negative in third, fourth, and fifth grade. For working memory (reversed-numbers test), the *g* × *sca* loadings were significant and positive for kindergarten entry, kindergarten spring, and fifth grade. The remaining coefficients for working memory were not statistically significant. In sum, of the 34 estimates, six were positive, five were not significantly different from zero, and 23 were negative. Figures S2 through S6 in the Supplemental Material display the *g* × *sca* interactions for the five domains.

In the model that included autoregressive paths conducted as a robustness check (see Table S2 in the Supplemental Material), the autoregressive paths were statistically significant and positive for mathematics, reading, science, and working memory (reversed-numbers task), with standardized estimates ranging from .08 to .46. For cognitive flexibility (card-sort task), not all autoregressive paths were statistically significant; however, as the scoring scheme changed between first and second grade for this task, the autoregressive-path model was difficult to interpret (Tourangeau et al., 2017). Both the *g* and *sca* loadings were, in general, smaller than in the model without autoregressive paths. Differences from the results of the main analysis emerged, for example, for cognitive flexibility (card-sort task), for which the *sca* loadings were not statistically significant for several waves. The patterns of *g × g* and *g* × *sca* interactions largely mirrored those from the main analysis.^1^

## Discussion

We analyzed data from 17,797 children to investigate ability and age differentiation in general cognitive abilities, age differentiation in specific abilities, and the interplay between domain-general and domain-specific abilities. We view age differentiation in specific abilities as a critical missing piece in current research on the structure of cognitive abilities.

Replicating previous findings (Breit et al., 2021; Tucker-Drob, 2009), our results supported ability differentiation in *g* in a large, longitudinal data set. There was no support for age differentiation in *g* (Breit et al., 2022); instead, we observed a consistent effect of *g* across this developmental period. However, the most striking loading patterns concerned the domain-specific factor loadings. For mathematics, reading, cognitive flexibility, and working memory, the *sca* loadings were small and, for some domains, even close to zero at kindergarten entry but increased over the course of childhood.

Theoretically, our results indicate that age differentiation might reasonably be conceptualized as qualitatively different from ability differentiation, with the former pertaining to specific abilities and the latter to general cognitive ability. Hence, the focus on general abilities in previous research on age differentiation likely obfuscated the most likely source of age differentiation, as well as its properties and manifestations. The increases in *sca* loadings between kindergarten and fourth grade for most tests instead indicate that content- or subject-domain-specific variance, and not *g*, systematically changes during this developmental period.

Our study’s findings regarding *sca* loadings also add to existing theories. Expanding the scope of differentiation research to include age differentiation in specific abilities better captures predictions from investment theory, as we looked at “intellectual investment where it happens” (see Ackerman, 2023, for similar arguments regarding adult intelligence). When children enter kindergarten, *g* alone may explain nearly all the covariance among domain-specific tests (e.g., math or reading ability). As children develop and learn from their environments, including the school environment, cognitive abilities may gradually differentiate (e.g., into more math-specific and reading-specific domains). For the executive-function measures—the card-sort and reversed-numbers tasks, which showed the same patterns of *sca* loadings as reading and math—additional explanations include changes in strategy as children develop through this period (Doebel & Zelazo, 2015). A further possibility, reconciling investment theory and mutualism, is that mutually beneficial couplings between executive-functioning skills, school-specific reading and mathematics skills, and respective investments translate into similar structural trajectories in *sca* loadings in childhood.

Interestingly, across different tasks, the *sca* loadings consistently dropped in the fifth-grade assessment, although the decrease was not statistically significant. Grade 5 coincides with the onset of preadolescence, which may be accompanied by decreases in learning motivation (Scherrer & Preckel, 2019) or shifts in developmental priorities (Demetriou et al., 2022). Of course, these interpretations remain speculative and warrant systematic attention in future research.

The results also provided some support for the circumvention-of-limits hypothesis (Hambrick & Meinz, 2011) for reading, science, the card-sort task, and, in later waves, mathematics. Hence, in most domains, children with lower general cognitive abilities could overcome this limitation to some extent through high levels of domain-specific expertise. Our analytical approach, the age group, and the large sample may have provided a viable context for detecting such compensatory mechanisms. The findings are also important from an applied angle, because promoting domain-specific skills can become an equalizing force for children with lower levels of general cognitive ability.

We acknowledge several limitations. Although our study followed children from kindergarten entry to fifth grade, it would have been desirable to investigate changes in both *g* and *sca* loadings in samples with larger age ranges, ideally from childhood to (late) adulthood. Such investigations will be key to building a strong theory of the development of domain-specific variance across the life span. Furthermore, our findings are based on a sample of children from the United States, which may limit the generalizability of the results. Also, we did not account for genetic and environmental influences and respective mechanisms (e.g., amplification; Briley & Tucker-Drob, 2013). In addition, changing *sca* loadings across waves may reflect some combination of measurement error and changes in the influence of latent *sca.*

Our study differs from previous factor-analytic differentiation research because of our focus on specific ability factor loadings (in addition to *g* loadings) as well as the chosen analytical approach (bifactor model); hence, the results are not directly comparable. Analytical choices (e.g., bifactor model versus a higher-order model) influence our understanding of the nature of cognitive abilities, and specificities of the bifactor model need to be kept in mind when interpreting our findings—for example, specific cognitive-ability factors are residuals, thus prioritizing *g*; *g* and specific cognitive-ability factors are independent; *g* has a direct influence on measured variables (Beaujean, 2015; Molenaar et al., 2010). Even though the bifactor model fit the purpose of our study, which aimed to gain insights into structural changes in both *g* and specific abilities well, we acknowledge that there are different and diverging theoretical viewpoints on the structure of cognitive abilities that are represented in different statistical models. Moreover, one difference between our study and previous work is that the specific cognitive-ability factors we modeled were measured by the same test across time. One possible explanation for our findings is that the specific cognitive abilities that become most differentiated across development are highly specific; for example, investing more or less practice in a particular kind of spatial activity may lead one spatial task, but not necessarily others, to become differentiated across development. If so, it would follow that age differentiation should be easier to observe when more specific skills are being tested.

Another important drawback is that we used only a single test to measure each domain-specific construct. Thus, we cannot separate test-specific variance from other sources of variance. Further work is needed to identify these origins of variance more precisely. Nevertheless, by demonstrating the value of adopting a specific ability lens for viewing children’s cognitive differentiation, our study suggests that the next wave in differentiation research may very well be domain specific.

## Supplemental Material

sj-docx-1-pss-10.1177_09567976251321382 – Supplemental material for Differentiation in Cognitive Abilities Beyond g: The Emergence of Domain-Specific Variance in Childhood Supplemental material, sj-docx-1-pss-10.1177_09567976251321382 for Differentiation in Cognitive Abilities Beyond g: The Emergence of Domain-Specific Variance in Childhood by Lisa Bardach, Robert Kalinowski and Drew H. Bailey in Psychological Science

sj-docx-2-pss-10.1177_09567976251321382 – Supplemental material for Differentiation in Cognitive Abilities Beyond g: The Emergence of Domain-Specific Variance in Childhood Supplemental material, sj-docx-2-pss-10.1177_09567976251321382 for Differentiation in Cognitive Abilities Beyond g: The Emergence of Domain-Specific Variance in Childhood by Lisa Bardach, Robert Kalinowski and Drew H. Bailey in Psychological Science

## Transparency

*Action Editor:* Evie Vergauwe

*Editor:* Simine Vazire

*Author Contributions*

**Lisa Bardach:** Conceptualization, Formal analysis, Methodology, Writing – original draft.

**Robert Kalinowski:** Conceptualization, Formal analysis, Methodology, Writing – original draft.

**Drew H. Bailey:** Conceptualization, Methodology, Supervision, Writing – review & editing.

**Artificial Intelligence:** No artificial-intelligence-assisted technologies were used in this research or the creation of this article.

**Ethics:** This article uses secondary data provided by the U.S. Department of Education. The analysis was registered with the institutional review board (IRB) at the University of California, Irvine, under IRB #5219.

**Open Practices:** No aspects of the study were preregistered. The materials consist of items adapted from several copyrighted assessment batteries, which are not publicly available because of copyright restrictions. More information and instructions for accessing these materials are available at the Open Science Framework, or OSF (https://osf.io/brdyt). Our study uses publicly available data from the ECLS-K:2011 study, and we share the data set we analyzed at the OSF (https://osf.io/brdyt). All analysis scripts are publicly available at the OSF (https://osf.io/brdyt). The computational reproducibility of the results has been independently confirmed by the journal’s STAR team.

## Notes

1. In addition to the analyses reported in our manuscript, we ran the following models: We set up another nonlinear confirmatory-factor-analysis model to address our research questions in a slightly different way (by including interactions between *g* and age, and *sca* and age). However, this model did not converge. In addition, we estimated our main nonlinear confirmatory factor analysis. model with constraints (linearly increasing factor loadings; the first and last loading were fixed and the rest freely estimated), but also encountered convergence problems.
