# Supplementary material for: Differentiation in Cognitive Abilities Beyond g: The Emergence of Domain-Specific Variance in Childhood
Source: Psychol Sci. 2025 Mar 18;36(3):168–83. doi: 10.1177/09567976251321382 (PMC13428966; doi:10.1177/09567976251321382)
Supplement: sj-docx-1-pss-10.1177_09567976251321382 – Supplemental material for Differentiation in Cognitive Abilities Beyond g: The Emergence of Domain-Specific Variance in Childhood [file sj-docx-1-pss-10.1177_09567976251321382.docx]

**Figure 1**

*Bivariate Correlations Between All Variables*


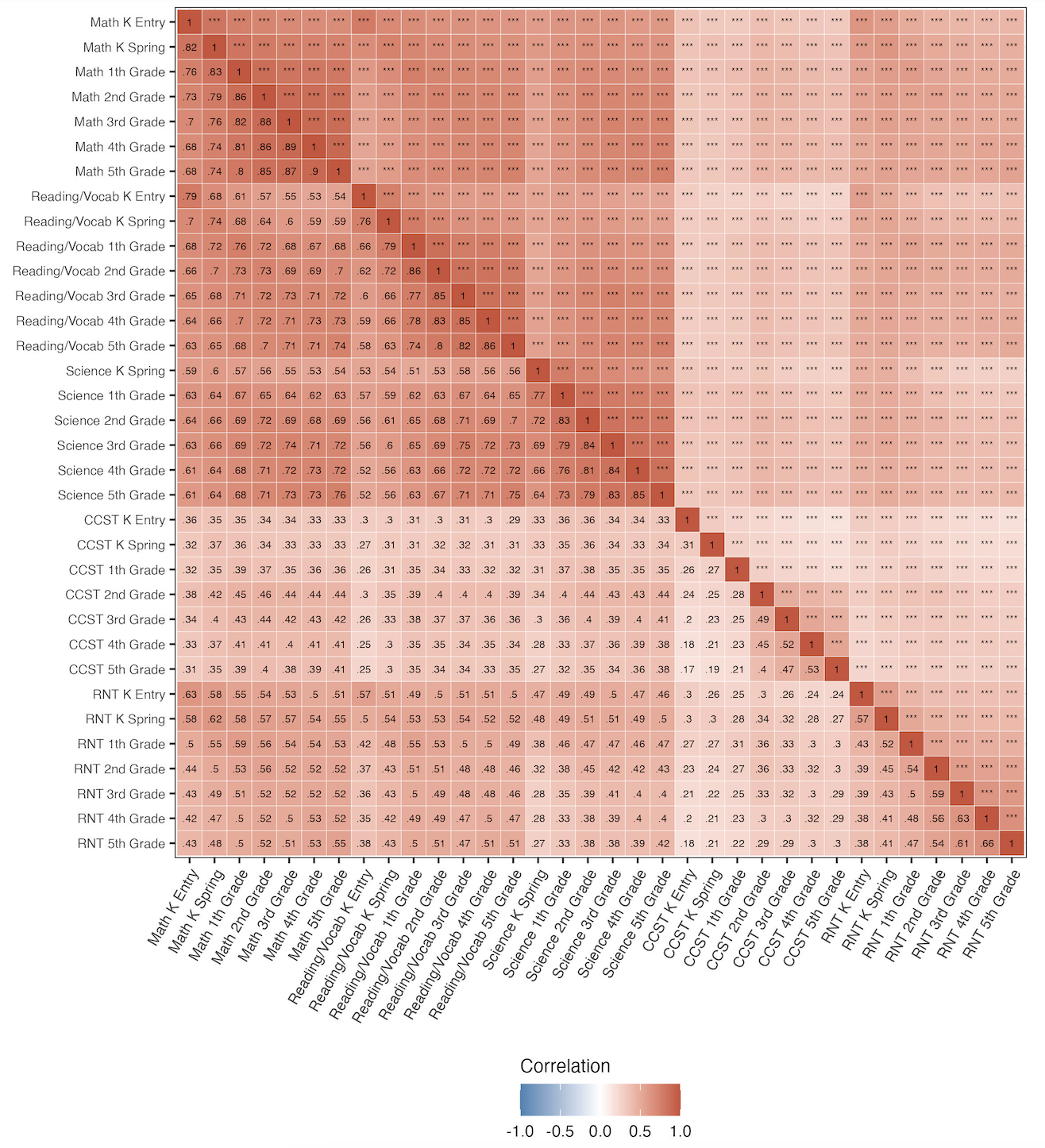


*Note.* Correlation coefficients are below and information regarding statistical significance
(* < .05, ** <.01, *** < .001) can be found above the diagonal.

**Figure S2**

*Interactions Between General Cognitive Abilities and Specific Cognitive Abilies for Mathematics*


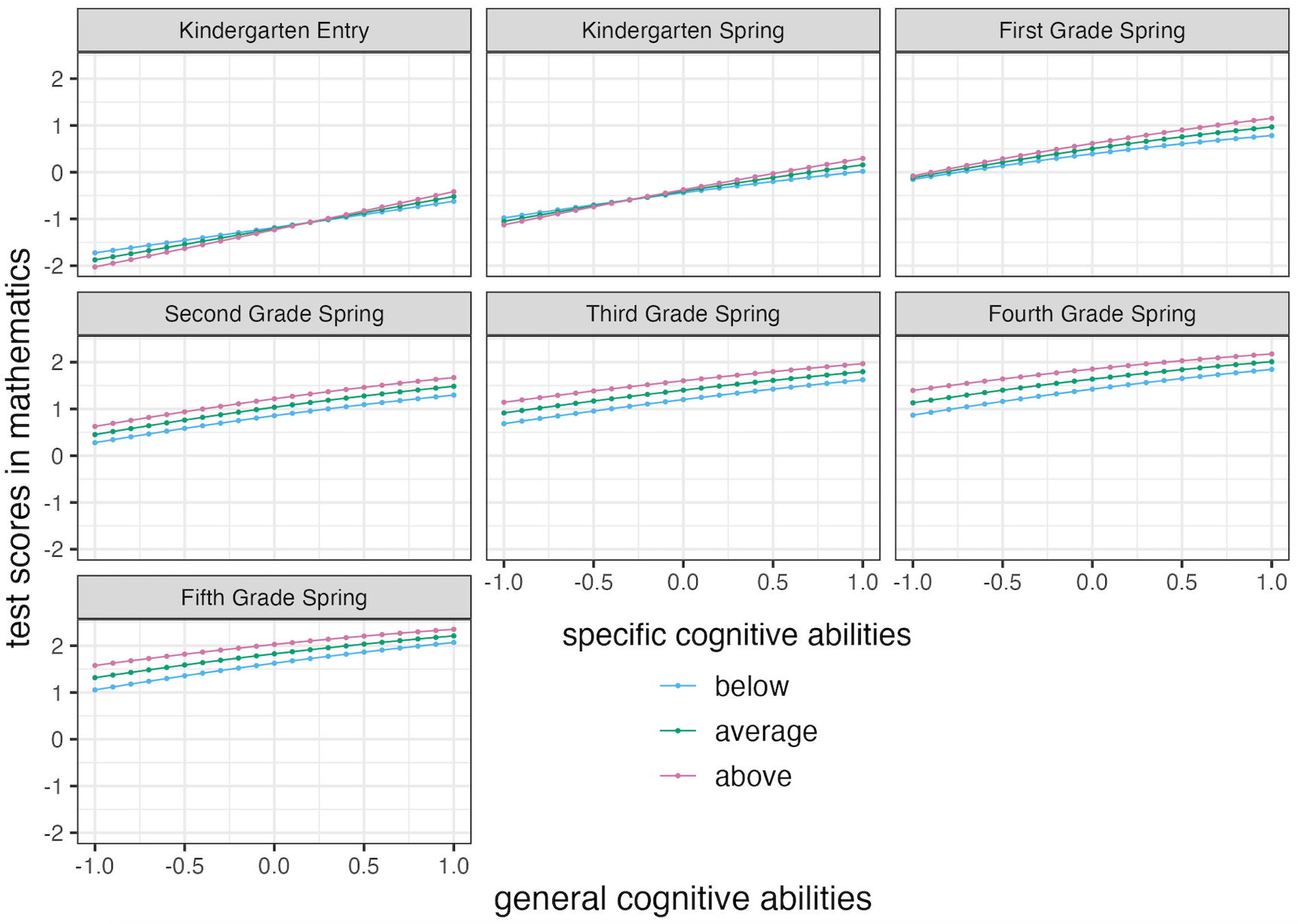


**Figure S3**

*Interactions Between General Cognitive Abilities and Specific Cognitive Abilies for Reading*

*
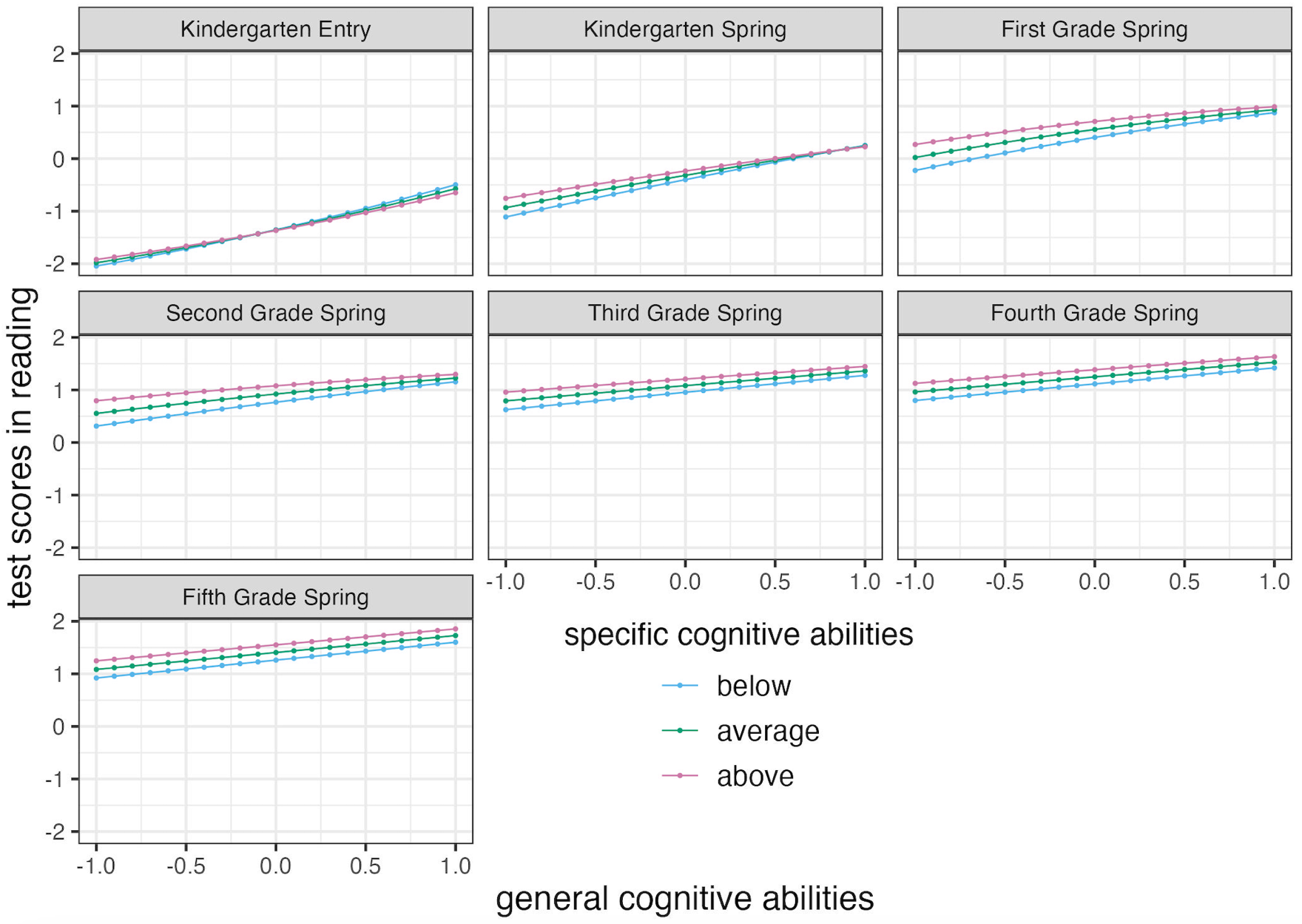
*

**Figure S4**

*Interactions Between General Cognitive Abilities and Specific Cognitive Abilities for Science*

*
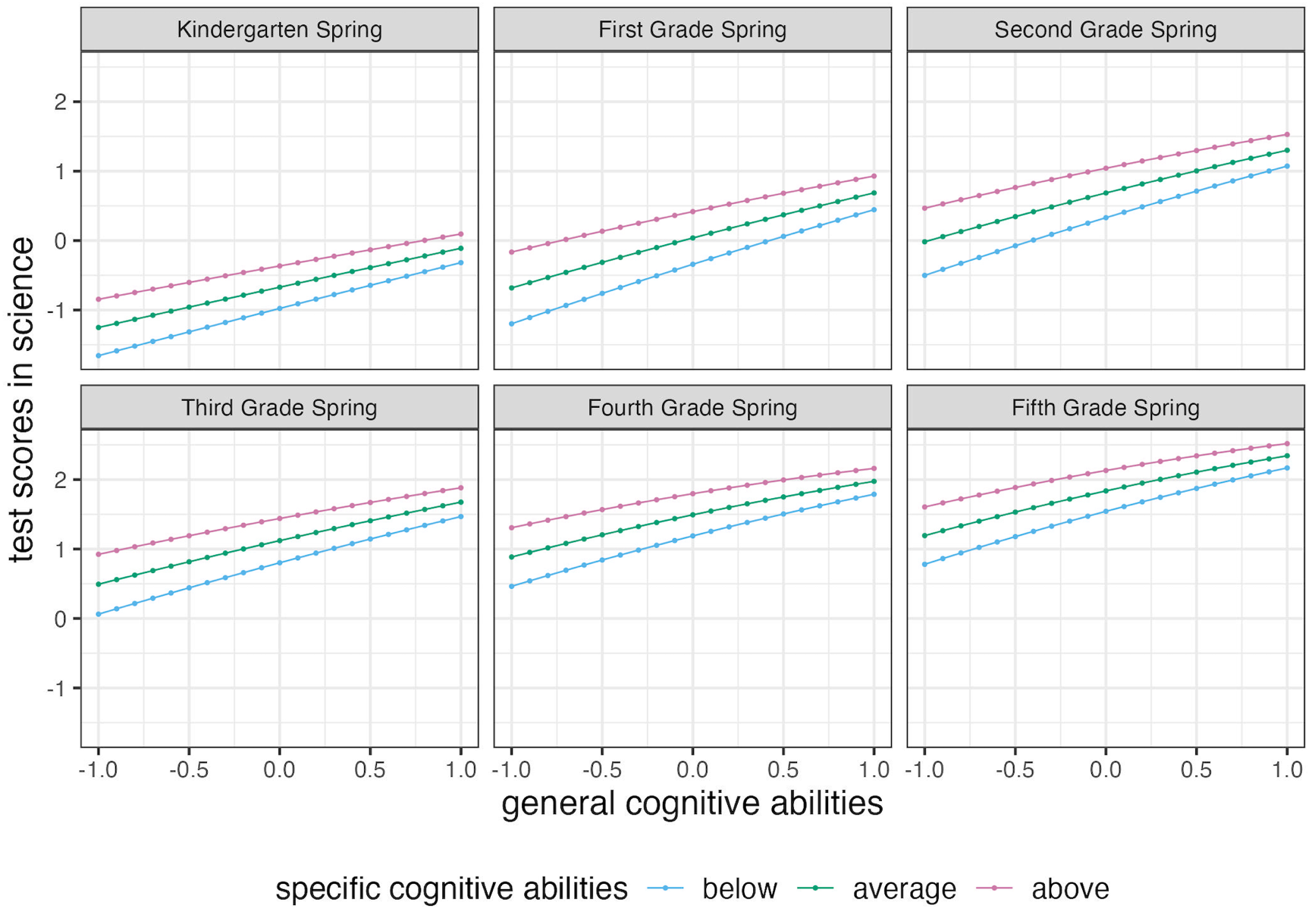
*

**Figure S5**

*Interactions Between General Cognitive Abilities and Specific Cognitive Abilities for Cognitive Flexibility*

*
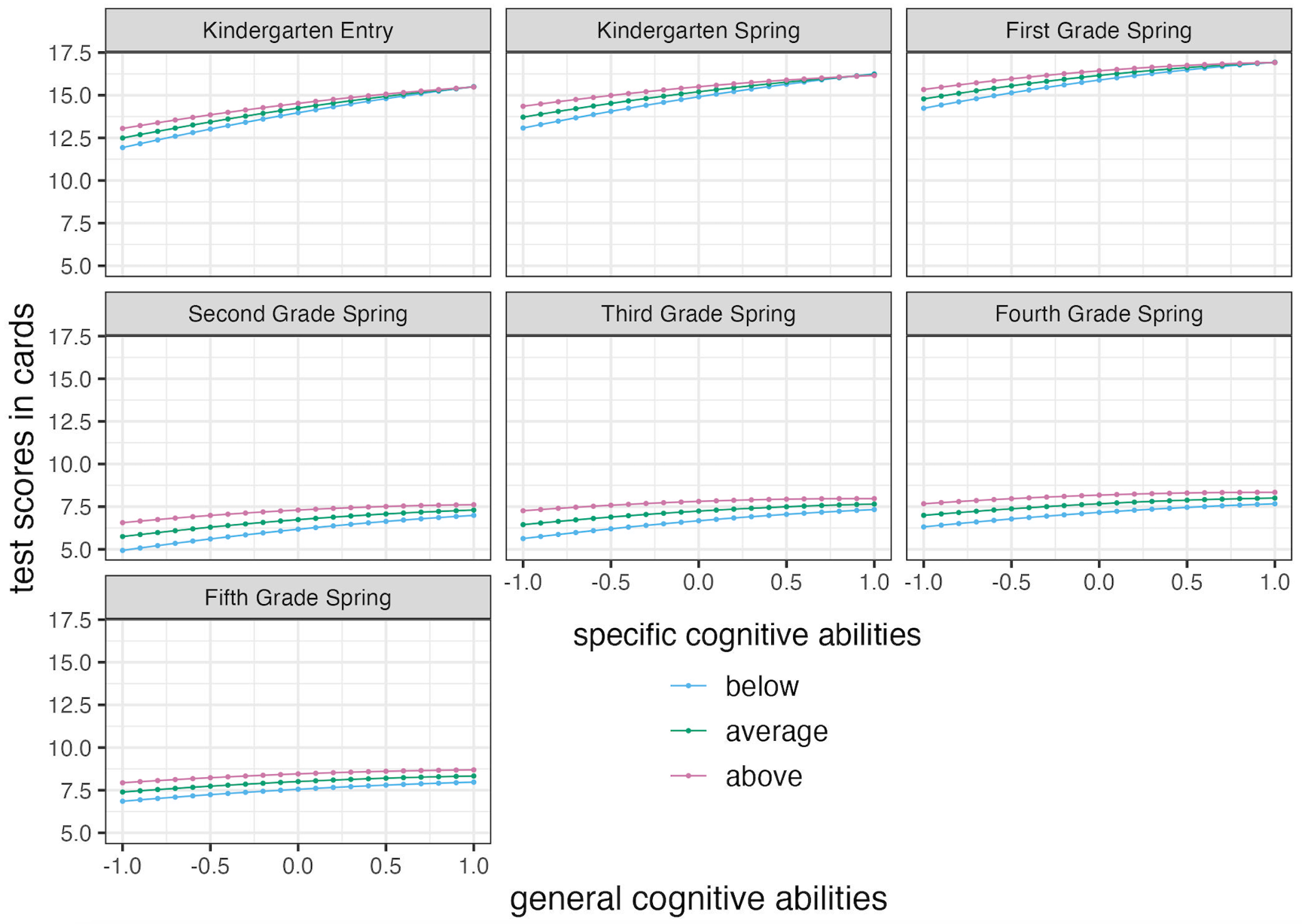
*

**Figure S6**

*Interactions Between General Cognitive Abilities and Specific Cognitive Abilities for Working Memory*

**
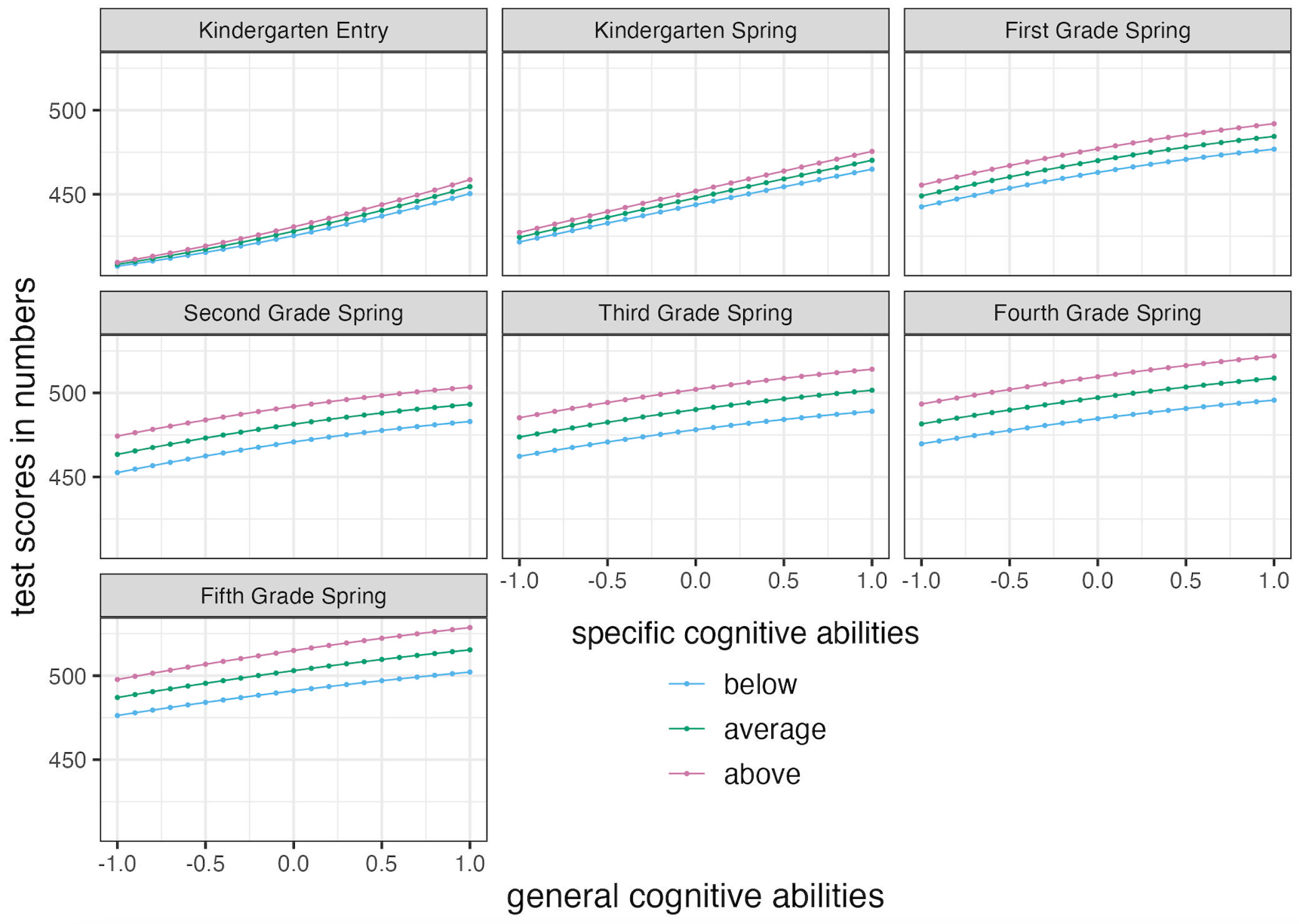
**
