# Supplementary material for: Differentiation in Cognitive Abilities Beyond g: The Emergence of Domain-Specific Variance in Childhood
Source: Psychol Sci. 2025 Mar 18;36(3):168–83. doi: 10.1177/09567976251321382 (PMC13428966; doi:10.1177/09567976251321382)
Supplement: sj-docx-2-pss-10.1177_09567976251321382 – Supplemental material for Differentiation in Cognitive Abilities Beyond g: The Emergence of Domain-Specific Variance in Childhood [file sj-docx-2-pss-10.1177_09567976251321382.docx]

**Table S1**

*Descriptive Statistics for All Variables*

| Variable | *M* | *SD* | *Min.* | *Max.* | *Missing* |
| --- | --- | --- | --- | --- | --- |
| Math kindergarten entry | -1.129 | 0.703 | -3.679 | 2.182 | 2,384 |
| Math kindergarten spring | -0.378 | 0.622 | -3.783 | 1.656 | 836 |
| Math first grade spring | 0.504 | 0.564 | -3.544 | 2.371 | 2,876 |
| Math second grade spring | 1.044 | 0.54 | -2.406 | 2.376 | 4,149 |
| Math third grade spring | 1.42 | 0.469 | -0.582 | 2.675 | 5,113 |
| Math fourth grade spring | 1.641 | 0.476 | -1.643 | 2.675 | 5,899 |
| Math fifth grade spring | 1,838 | 0.478 | -1.56 | 2.681 | 6,553 |
| Reading kindergarten entry | -1.223 | 0.798 | -3.769 | 2.991 | 2,310 |
| Reading kindergarten spring | -0.282 | 0.668 | -4.254 | 1.351 | 793 |
| Reading first grade spring | 0.547 | 0.501 | -3.994 | 1.466 | 2,864 |
| Reading second grade spring | 0.94 | 0.369 | -1.9 | 1.641 | 4,142 |
| Reading third grade spring | 1.116 | 0.306 | -0.261 | 2.033 | 5,113 |
| Reading fourth grade spring | 1.285 | 0.303 | 0.065 | 1.978 | 5,905 |
| Reading fifth grade spring | 1.452 | 0.354 | 0.025 | 2.206 | 6,552 |
| Science kindergarten spring | -0.607 | 0.741 | -2.65 | 0.991 | 1,043 |
| Science first grade spring | 0.095 | 0.806 | -2.963 | 1.955 | 2,907 |
| Science second grade spring | 0.731 | 0.754 | -3.29 | 2.612 | 4,160 |
| Science third grade spring | 1.164 | 0.667 | -1.643 | 2.678 | 5,123 |
| Science fourth grade spring | 1.51 | 0.638 | -2.004 | 2.726 | 5,910 |
| Science fifth grade spring | 1.855 | 0.676 | -1.382 | 2.784 | 6,560 |
| Change card sort task kindergarten entry | 14.203 | 3.329 | 0 | 18 | 2,375 |
| Change card sort task kindergarten spring | 15.145 | 2.796 | 0 | 18 | 830 |
| Change card sort task first grade spring | 16.048 | 2.313 | 0 | 18 | 2,870 |
| Change card sort task second grade spring | 6.689 | 1.354 | 1.625 | 10 | 4,205 |
| Change card sort task third grade spring | 7.189 | 1.107 | 1.75 | 10 | 5,235 |
| Change card sort task fourth grade spring | 7.617 | 0.983 | 1.875 | 10 | 5,958 |
| Change card sort task fifth grade spring | 7.968 | 0.55 | 2 | 10 | 6,593 |
| Reversed numbers task kindergarten entry | 433.006 | 30.21 | 393 | 581 | 2,381 |
| Reversed numbers task kindergarten spring | 449.677 | 30.517 | 393 | 572 | 832 |
| Reversed numbers task first grade spring | 469.33 | 25.816 | 393 | 596 | 2,872 |
| Reversed numbers task second grade spring | 480.683 | 23.278 | 403 | 581 | 4,147 |
| Reversed numbers task third grade spring | 489.804 | 22.213 | 403 | 603 | 5,102 |
| Reversed numbers task fourth grade spring | 497.244 | 21.698 | 403 | 588 | 5,894 |
| Reversed numbers task fifth grade spring | 503.31 | 22.359 | 403 | 588 | 6,549 |

**Table S2**

*Standardized Parameter Estimates for the Model Including Autoregressive Paths: Age Differentiation in g (Longitudinal Comparisons of g-Loadings by Age), Age Differentiation in Specific Skills (Longitudinal Comparison of sca-Loadings by Age), Ability Differentiation (gxg Interactions), Tests of the Circumvention-of-Limits Hypothesis (gxsca Interactions), and Autoregressive Effects*

|  |  | Model Parameters | | | | | | |  |  |
| --- | --- | --- | --- | --- | --- | --- | --- | --- | --- | --- |
| Tested Domain |  | Kindergarten Entry | Kindergarten Spring | First Grade Spring | Second Grade Spring | Third Grade Spring | Fourth Grade Spring | Fifth Grade Spring |  |  |
| Math | |  |  |  |  |  |  |  |  |  |
| *g* |  | **0.853 (0.004)** | **0.572 (0.013)** | **0.686 (0.012)** | **0.662 (0.012)** | **0.697 (0.022)** | **0.651 (0.028)** | **0.626 (0.027)** |  |  |
| *sca* |  | -0.012 (0.012) | **0.075 (0.008)** | **0.139 (0.009**) | **0.232 (0.010)** | **0.279 (0.015)** | **0.256 (0.017)** | **0.235 (0.015)** |  |  |
| *gxg* |  | 0.014 (0.007) | **-0.070 (0.005)** | **-0.118 (0.006)** | **-0.090 (0.006)** | **-0.077 (0.007)** | **-0.115 (0.009)** | **-0.096 (0.009)** |  |  |
| *gxsca* |  | **0.143 (0.011)** | **0.080 (0.012)** | **0.094 (0.015)** | **-0.030 (0.011)** | **-0.058 (0.008)** | **-0.061 (0.010)** | **-0.088 (0.011)** |  |  |
| *W-1* 🡪 *W* |  |  | **0.344 (0.014)** | **0.216 (0.013)** | **0.246 (0.014)** | **0.193 (0.026)** | **0.232 (0.031)** | **0.273 (0.030)** |  |  |
| Reading/Vocab | |  |  |  |  |  |  |  |  |  |
| *g* |  | **0.779 (0.007)** | **0.430 (0.012)** | **0.577 (0.015)** | **0.604(0.025)** | **0.700 (0.038)** | **0.645 (0.034)** | **0.525 (0.027)** |  |  |
| *sca* |  | -0.011 (0.019) | **0.098 (0.011)** | **0.203 (0.016)** | **0.282 (0.011)** | **0.214 (0.036)** | **0.241 (0.017)** | **0.162 (0.023)** |  |  |
| *gxg* |  | **0.076 (0.006)** | **-0.098 (0.005)** | **-0.151 (0.007)** | **-0.051 (0.008)** | -0.008 (0.006) | **-0.023 (0.005)** | 0.004 (0.004) |  |  |
| *gxsca* |  | **-0.045 (0.013)** | **-0.070 (0.016)** | **-0.114 (0.018)** | **-0.185 (0.011)** | **-0.104 (0.013)** | **-0.063 (0.016)** | **-0.022 (0.008)** |  |  |
| *W-1* 🡪 *W* |  |  | **0.460 (0.014)** | **0.309 (0.018)** | **0.276 (0.030)** | **0.190 (0.045)** | **0.243 (0.039)** | **0.381 (0.031)** |  |  |
| Science | |  |  |  |  |  |  |  |  |  |
| *g* |  | † | **0.705 (0.006)** | **0.539 (0.009)** | **0.587 (0.013)** | **0.723 (0.018)** | **0.664 (0.025)** | **0.589 (0.020)** |  |  |
| *sca* |  | † | **0.294 (0.010)** | **0.237 (0.009)** | **0.254 (0.011)** | **0.338 (0.013)** | **0.308 (0.021)** | **0.247 (0.018)** |  |  |
| *gxg* |  | † | -0.007 (0.008) | **-0.033 (0.006)** | **-0.037 (0.006)** | **-0.034 (0.008)** | **-0.079 (0.007)** | **-0.069 (0.006)** |  |  |
| *gxsca* |  | † | **-0.141 (0.010)** | **-0.135 (0.010)** | **-0.141 (0.012)** | **-0.165 (0.010)** | **-0.167 (0.015)** | **-0.128 (0.013)** |  |  |
| *W-1* 🡪 *W* |  |  |  | **0.333 (0.011)** | **0.284 (0.016)** | **0.122 (0.020)** | **0.163 (0.030)** | **0.269 (0.026)** |  |  |
| Change Card Sort Task | | |  |  |  |  |  |  |  |  |
| *g* |  | **0.425 (0.010)** | **0.357 (0.012)** | **0.401 (0.014)** | **0.528 (0.028)** | **0.408 (0.035)** | **0.316 (0.020)** | **0.279 (0.015)** |  |  |
| *sca* |  | 0.097 (0.045) | **0.099 (0.031)** | 0.150 (0.075) | **0.367 (0.035)** | 0.197 (0.087) | **0.137 (0.023)** | **0.101 (0.033)** |  |  |
| *gxg* |  | **-0.073 (0.010)** | **-0.078 (0.008)** | **-0.123 (0.012)** | **-0.152 (0.014)** | **-0.155 (0.011)** | **-0.124 (0.011)** | **-0.083 (0.012)** |  |  |
| *gxsca* |  | -0.121 (0.047) | **-0.128 (0.035)** | -0.172 (0.072) | **-0.340 (0.020)** | **-0.278 (0.085)** | **-0.181 (0.044)** | **-0.169 (0.040)** |  |  |
| *W-1* 🡪 *W* |  |  | **0.146 (0.018)** | **0.077 (0.024)** | -0.036 (0.056) | 0.131 (0.072) | **0.290 (0.040)** | **0.352 (0.028)** |  |  |
| Reversed Numbers Task | |  |  |  |  |  |  |  |  |  |
| *g* |  | **0.700 (0.005)** | **0.533 (0.010)** | **0.540 (0.016)** | **0.408 (0.018)** | **0.370 (0.021)** | **0.350 (0.014)** | **0.348 (0.012)** |  |  |
| *sca* |  | **0.123 (0.020)** | **0.090 (0.026)** | **0.244 (0.069)** | -0.085 (0.034) | **0.235 (0.034)** | 0.020 (0.049) | **0.154 (0.025)** |  |  |
| *gxg* |  | **0.102 (0.007)** | **-0.046 (0.006)** | **-0.132 (0.006)** | **-0.098 (0.009)** | **-0.067 (0.008)** | **-0.049 (0.009)** | **-0.044 (0.008)** |  |  |
| *gxsca* |  | **0.061 (0.013)** | 0.012 (0.011) | -0.024 (0.047) | **0.332 (0.023)** | 0.065 (0.052) | **0.191 (0.025)** | **0.149 (0.026)** |  |  |
| *W-1* 🡪 *W* |  |  | **0.231 (0.013)** | **0.137 (0.025)** | **0.294 (0.026)** | **0.356 (0.037)** | **0.401 (0.023)** | **0.418 (0.017)** |  |  |

*Note.* Values in bold indicate *p <* .01. Standard errors are given in parentheses. Errors were clustered at the first-grade school level.
*g =* the first extracted factor (the general factor); *sca =* the second factor, uncorrelated with *g* (the specific skill factor unique to each tested domain); *gxg* = the *g*-by-*g* interaction; *gxs* = the *g*-by-*sca* interaction, with the *sca*-factor varying by the tested domain. W-1 🡪 W = autoregressive paths (within content domains) added as a robustness check. The unusual loadings within the Dimensional Change Card Sort Task may be due to the scoring scheme changing between first and second grade, thus making the autoregressive path model difficult to interpret (Tourangeau et. al., 2017).
